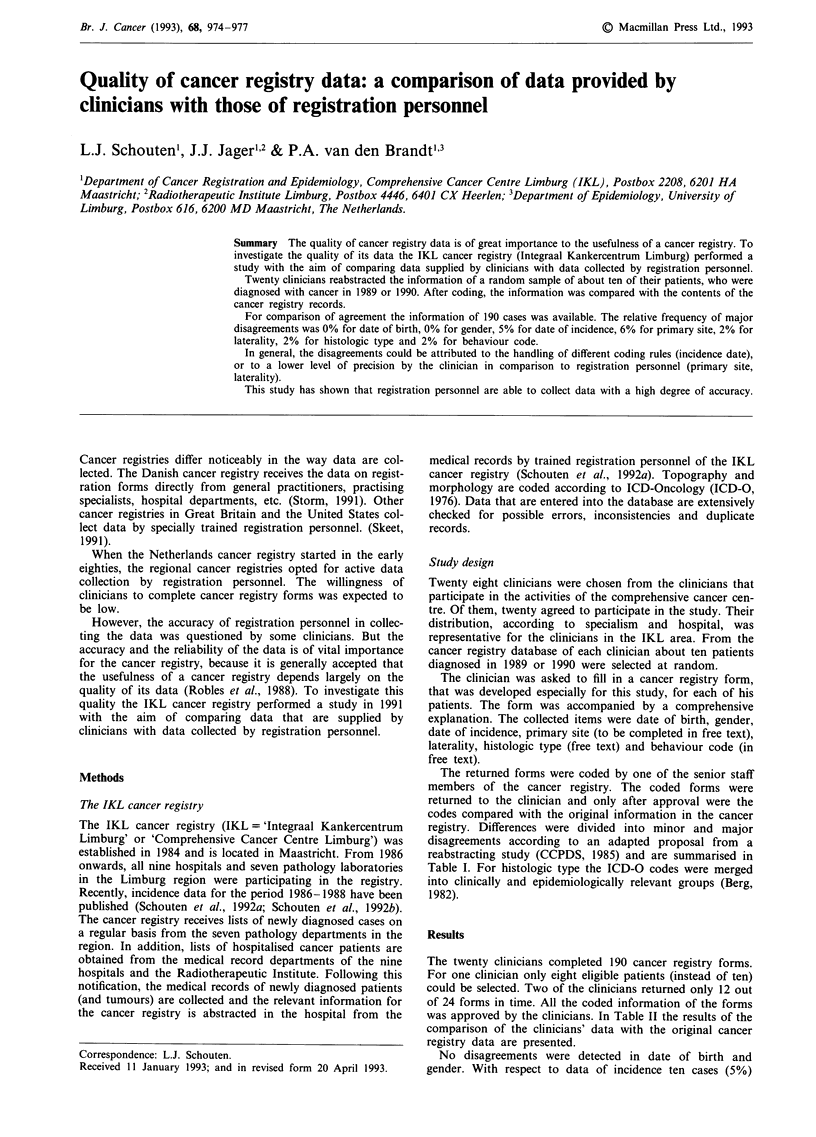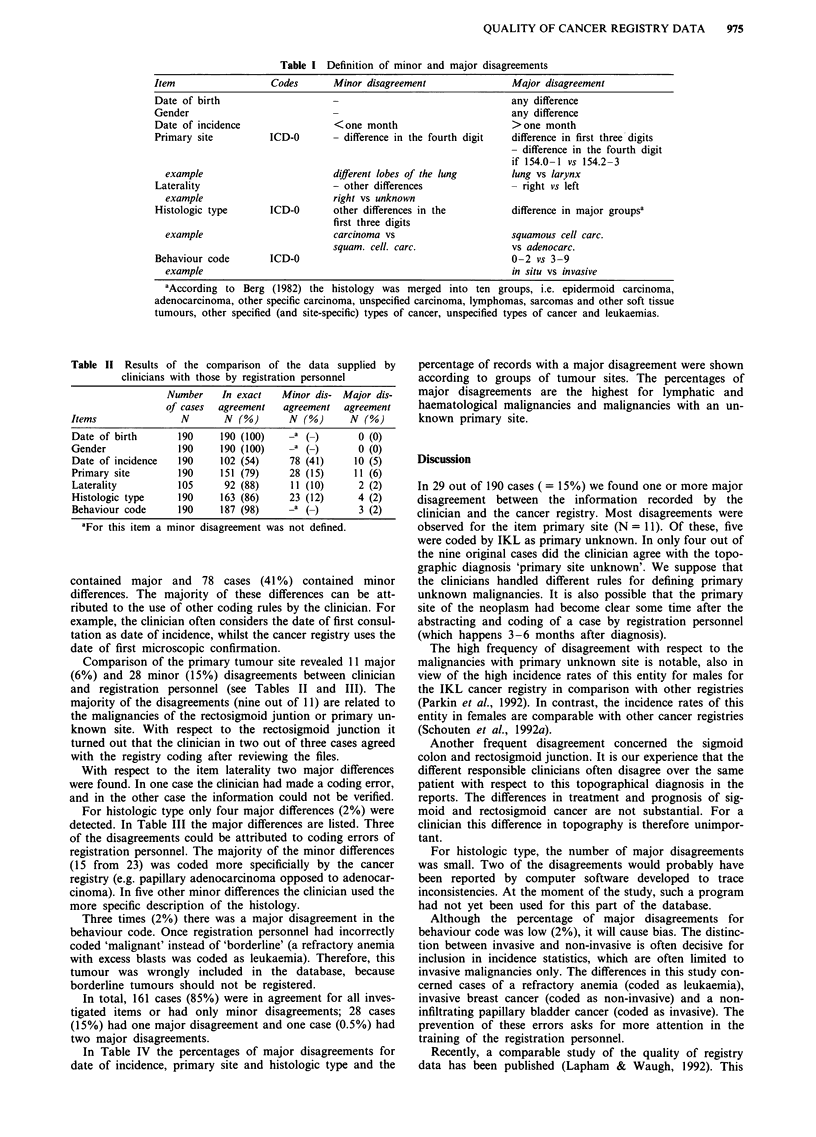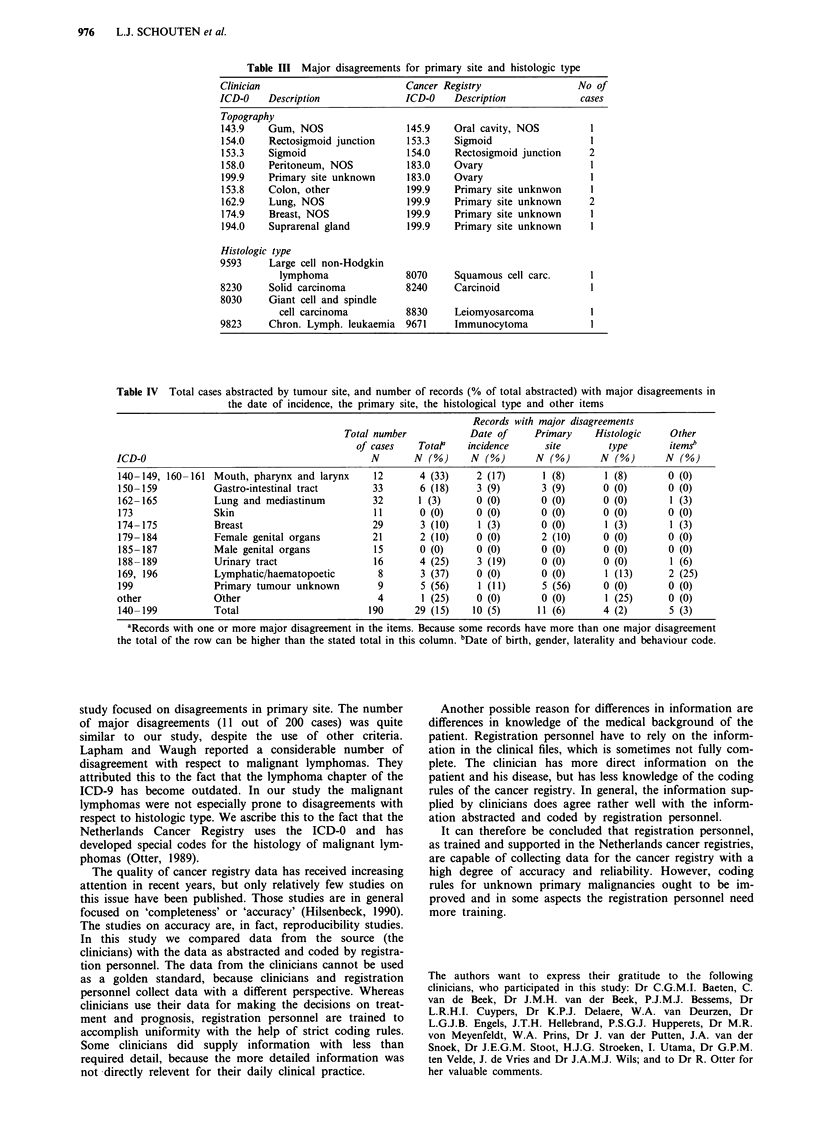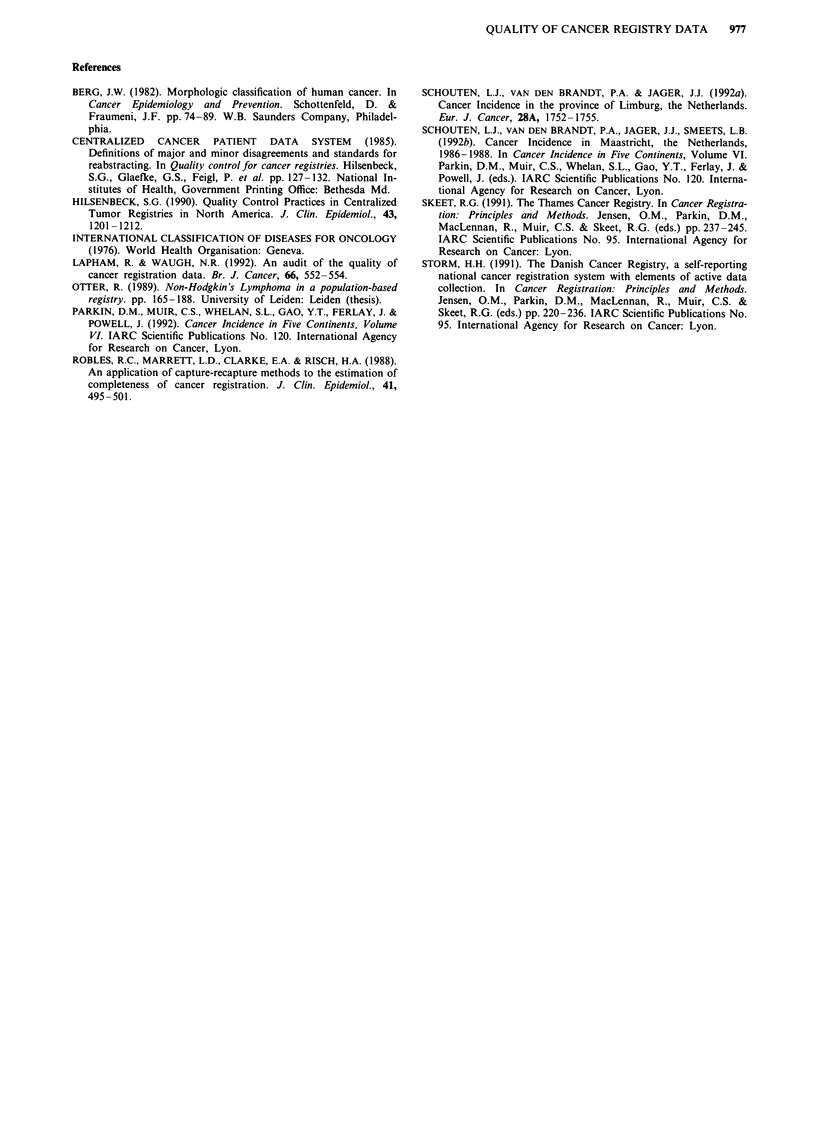# Quality of cancer registry data: a comparison of data provided by clinicians with those of registration personnel.

**DOI:** 10.1038/bjc.1993.464

**Published:** 1993-11

**Authors:** L. J. Schouten, J. J. Jager, P. A. van den Brandt

**Affiliations:** Department of Cancer Registration and Epidemiology, Comprehensive Cancer Centre Limburg (IKL), Maastricht, The Netherlands.

## Abstract

The quality of cancer registry data is of great importance to the usefulness of a cancer registry. To investigate the quality of its data the IKL cancer registry (Integraal Kankercentrum Limburg) performed a study with the aim of comparing data supplied by clinicians with data collected by registration personnel. Twenty clinicians reabstracted the information of a random sample of about ten of their patients, who were diagnosed with cancer in 1989 or 1990. After coding, the information was compared with the contents of the cancer registry records. For comparison of agreement the information of 190 cases was available. The relative frequency of major disagreements was 0% for date of birth, 0% for gender, 5% for date of incidence, 6% for primary site, 2% for laterality, 2% for histologic type and 2% for behaviour code. In general, the disagreements could be attributed to the handling of different coding rules (incidence date), or to a lower level of precision by the clinician in comparison to registration personnel (primary site, laterality). This study has shown that registration personnel are able to collect data with a high degree of accuracy.


Br. J. Cancer (1993), 68, 974-977 © Macmillan Press Ltd., 1993

### Quality of cancer registry data: a comparison of data provided by clinicians with those of registration personnel

L.J. Schouten[1], J.J. Jager[1,2] & P.A. van den Brandt[1,3]

[1]*Department of Cancer Registration and Epidemiology, Comprehensive Cancer Centre Limburg (IKL), Postbox 2208, 6201 HA Maastricht;* [2]*Radiotherapeutic Institute Limburg, Postbox 4446, 6401 CX Heerlen;* [3]*Department of Epidemiology, University of Limburg, Postbox 616, 6200 MD Maastricht, The Netherlands.*

**Summary** The quality of cancer registry data is of great importance to the usefulness of a cancer registry. To investigate the quality of its data the IKL cancer registry (Integraal Kankercentrum Limburg) performed a study with the aim of comparing data supplied by clinicians with data collected by registration personnel.

Twenty clinicians reabstracted the information of a random sample of about ten of their patients, who were diagnosed with cancer in 1989 or 1990. After coding, the information was compared with the contents of the cancer registry records.

For comparison of agreement the information of 190 cases was available. The relative frequency of major disagreements was 0% for date of birth, 0% for gender, 5% for date of incidence, 6% for primary site, 2% for laterality, 2% for histologic type and 2% for behaviour code.

In general, the disagreements could be attributed to the handling of different coding rules (incidence date), or to a lower level of precision by the clinician in comparison to registration personnel (primary site, laterality).

This study has shown that registration personnel are able to collect data with a high degree of accuracy.

Cancer registries differ noticeably in the way data are collected. The Danish cancer registry receives the data on registration forms directly from general practitioners, practising specialists, hospital departments, etc. (Storm, 1991). Other cancer registries in Great Britain and the United States collect data by specially trained registration personnel. (Skeet, 1991).

When the Netherlands cancer registry started in the early eighties, the regional cancer registries opted for active data collection by registration personnel. The willingness of clinicians to complete cancer registry forms was expected to be low.

However, the accuracy of registration personnel in collecting the data was questioned by some clinicians. But the accuracy and the reliability of the data is of vital importance for the cancer registry, because it is generally accepted that the usefulness of a cancer registry depends largely on the quality of its data (Robles *et al.*, 1988). To investigate this quality the IKL cancer registry performed a study in 1991 with the aim of comparing data that are supplied by clinicians with data collected by registration personnel.

### Methods

### *The IKL cancer registry*

The IKL cancer registry (IKL = 'Integraal Kankercentrum Limburg' or 'Comprehensive Cancer Centre Limburg') was established in 1984 and is located in Maastricht. From 1986 onwards, all nine hospitals and seven pathology laboratories in the Limburg region were participating in the registry. Recently, incidence data for the period 1986-1988 have been published (Schouten *et al.*, 1992*a*; Schouten *et al.*, 1992*b*). The cancer registry receives lists of newly diagnosed cases on a regular basis from the seven pathology departments in the region. In addition, lists of hospitalised cancer patients are obtained from the medical record departments of the nine hospitals and the Radiotherapeutic Institute. Following this notification, the medical records of newly diagnosed patients (and tumours) are collected and the relevant information for the cancer registry is abstracted in the hospital from the medical records by trained registration personnel of the IKL cancer registry (Schouten *et al.*, 1992*a*). Topography and morphology are coded according to ICD-Oncology (ICD-O, 1976). Data that are entered into the database are extensively checked for possible errors, inconsistencies and duplicate records.

### *Study design*

Twenty eight clinicians were chosen from the clinicians that participate in the activities of the comprehensive cancer centre. Of them, twenty agreed to participate in the study. Their distribution, according to specialism and hospital, was representative for the clinicians in the IKL area. From the cancer registry database of each clinician about ten patients diagnosed in 1989 or 1990 were selected at random.

The clinician was asked to fill in a cancer registry form, that was developed especially for this study, for each of his patients. The form was accompanied by a comprehensive explanation. The collected items were date of birth, gender, date of incidence, primary site (to be completed in free text), laterality, histologic type (free text) and behaviour code (in free text).

The returned forms were coded by one of the senior staff members of the cancer registry. The coded forms were returned to the clinician and only after approval were the codes compared with the original information in the cancer registry. Differences were divided into minor and major disagreements according to an adapted proposal from a reabstracting study (CCPDS, 1985) and are summarised in Table I. For histologic type the ICD-O codes were merged into clinically and epidemiologically relevant groups (Berg, 1982).

### Results

The twenty clinicians completed 190 cancer registry forms. For one clinician only eight eligible patients (instead of ten) could be selected. Two of the clinicians returned only 12 out of 24 forms in time. All the coded information of the forms was approved by the clinicians. In Table II the results of the comparison of the clinicians' data with the original cancer registry data are presented.

No disagreements were detected in date of birth and gender. With respect to data of incidence ten cases (5%)

Correspondence: L.J. Schouten.
Received 11 January 1993; and in revised form 20 April 1993.

**Table I** Definition of minor and major disagreements

| *Item* | *Codes* | *Minor disagreement* | *Major disagreement* |
|---|---|---|---|
| Date of birth | | – | any difference |
| Gender | | – | any difference |
| Date of incidence | | <one month | >one month |
| Primary site | ICD-0 | – difference in the fourth digit | difference in first three digits<br>– difference in the fourth digit<br>if 154.0–1 *vs* 154.2–3 |
| *example* | | *different lobes of the lung* | *lung* vs *larynx* |
| Laterality | | – other differences | – right *vs* left |
| *example* | | *right* vs *unknown* | |
| Histologic type | ICD-0 | other differences in the first three digits | difference in major groups[a] |
| *example* | | *carcinoma* vs *squam. cell. carc.* | *squamous cell carc.* vs *adenocarc.* |
| Behaviour code | ICD-0 | | 0–2 *vs* 3–9 |
| *example* | | | *in situ* vs *invasive* |

[a]According to Berg (1982) the histology was merged into ten groups, i.e. epidermoid carcinoma, adenocarcinoma, other specific carcinoma, unspecified carcinoma, lymphomas, sarcomas and other soft tissue tumours, other specified (and site-specific) types of cancer, unspecified types of cancer and leukaemias.

**Table II** Results of the comparison of the data supplied by clinicians with those by registration personnel

| *Items* | *Number of cases N* | *In exact agreement N (%)* | *Minor disagreement N (%)* | *Major disagreement N (%)* |
|---|---|---|---|---|
| Date of birth | 190 | 190 (100) | –[a] (–) | 0 (0) |
| Gender | 190 | 190 (100) | –[a] (–) | 0 (0) |
| Date of incidence | 190 | 102 (54) | 78 (41) | 10 (5) |
| Primary site | 190 | 151 (79) | 28 (15) | 11 (6) |
| Laterality | 105 | 92 (88) | 11 (10) | 2 (2) |
| Histologic type | 190 | 163 (86) | 23 (12) | 4 (2) |
| Behaviour code | 190 | 187 (98) | –[a] (–) | 3 (2) |

[a]For this item a minor disagreement was not defined.

contained major and 78 cases (41%) contained minor differences. The majority of these differences can be attributed to the use of other coding rules by the clinician. For example, the clinician often considers the date of first consultation as date of incidence, whilst the cancer registry uses the date of first microscopic confirmation.

Comparison of the primary tumour site revealed 11 major (6%) and 28 minor (15%) disagreements between clinician and registration personnel (see Tables II and III). The majority of the disagreements (nine out of 11) are related to the malignancies of the rectosigmoid juntion or primary unknown site. With respect to the rectosigmoid junction it turned out that the clinician in two out of three cases agreed with the registry coding after reviewing the files.

With respect to the item laterality two major differences were found. In one case the clinician had made a coding error, and in the other case the information could not be verified.

For histologic type only four major differences (2%) were detected. In Table III the major differences are listed. Three of the disagreements could be attributed to coding errors of registration personnel. The majority of the minor differences (15 from 23) was coded more specificially by the cancer registry (e.g. papillary adenocarcinoma opposed to adenocarcinoma). In five other minor differences the clinician used the more specific description of the histology.

Three times (2%) there was a major disagreement in the behaviour code. Once registration personnel had incorrectly coded 'malignant' instead of 'borderline' (a refractory anemia with excess blasts was coded as leukaemia). Therefore, this tumour was wrongly included in the database, because borderline tumours should not be registered.

In total, 161 cases (85%) were in agreement for all investigated items or had only minor disagreements; 28 cases (15%) had one major disagreement and one case (0.5%) had two major disagreements.

In Table IV the percentages of major disagreements for date of incidence, primary site and histologic type and the percentage of records with a major disagreement were shown according to groups of tumour sites. The percentages of major disagreements are the highest for lymphatic and haematological malignancies and malignancies with an unknown primary site.

### Discussion

In 29 out of 190 cases ( = 15%) we found one or more major disagreement between the information recorded by the clinician and the cancer registry. Most disagreements were observed for the item primary site (N = 11). Of these, five were coded by IKL as primary unknown. In only four out of the nine original cases did the clinician agree with the topographic diagnosis 'primary site unknown'. We suppose that the clinicians handled different rules for defining primary unknown malignancies. It is also possible that the primary site of the neoplasm had become clear some time after the abstracting and coding of a case by registration personnel (which happens 3–6 months after diagnosis).

The high frequency of disagreement with respect to the malignancies with primary unknown site is notable, also in view of the high incidence rates of this entity for males for the IKL cancer registry in comparison with other registries (Parkin *et al.*, 1992). In contrast, the incidence rates of this entity in females are comparable with other cancer registries (Schouten *et al.*, 1992*a*).

Another frequent disagreement concerned the sigmoid colon and rectosigmoid junction. It is our experience that the different responsible clinicians often disagree over the same patient with respect to this topographical diagnosis in the reports. The differences in treatment and prognosis of sigmoid and rectosigmoid cancer are not substantial. For a clinician this difference in topography is therefore unimportant.

For histologic type, the number of major disagreements was small. Two of the disagreements would probably have been reported by computer software developed to trace inconsistencies. At the moment of the study, such a program had not yet been used for this part of the database.

Although the percentage of major disagreements for behaviour code was low (2%), it will cause bias. The distinction between invasive and non-invasive is often decisive for inclusion in incidence statistics, which are often limited to invasive malignancies only. The differences in this study concerned cases of a refractory anemia (coded as leukaemia), invasive breast cancer (coded as non-invasive) and a non-infiltrating papillary bladder cancer (coded as invasive). The prevention of these errors asks for more attention in the training of the registration personnel.

Recently, a comparable study of the quality of registry data has been published (Lapham & Waugh, 1992). This

**Table III** Major disagreements for primary site and histologic type

| *Clinician ICD-0* | *Description* | *Cancer Registry ICD-0* | *Description* | *No of cases* |
|---|---|---|---|---|
| *Topography* | | | | |
| 143.9 | Gum, NOS | 145.9 | Oral cavity, NOS | 1 |
| 154.0 | Rectosigmoid junction | 153.3 | Sigmoid | 1 |
| 153.3 | Sigmoid | 154.0 | Rectosigmoid junction | 2 |
| 158.0 | Peritoneum, NOS | 183.0 | Ovary | 1 |
| 199.9 | Primary site unknown | 183.0 | Ovary | 1 |
| 153.8 | Colon, other | 199.9 | Primary site unknwon | 1 |
| 162.9 | Lung, NOS | 199.9 | Primary site unknown | 2 |
| 174.9 | Breast, NOS | 199.9 | Primary site unknown | 1 |
| 194.0 | Suprarenal gland | 199.9 | Primary site unknown | 1 |
| *Histologic type* | | | | |
| 9593 | Large cell non-Hodgkin lymphoma | 8070 | Squamous cell carc. | 1 |
| 8230 | Solid carcinoma | 8240 | Carcinoid | 1 |
| 8030 | Giant cell and spindle cell carcinoma | 8830 | Leiomyosarcoma | 1 |
| 9823 | Chron. Lymph. leukaemia | 9671 | Immunocytoma | 1 |

**Table IV** Total cases abstracted by tumour site, and number of records (% of total abstracted) with major disagreements in the date of incidence, the primary site, the histological type and other items

| | | | *Records with major disagreements* | | | | |
|---|---|---|---|---|---|---|---|
| *ICD-0* | | *Total number of cases N* | *Total[a] N (%)* | *Date of incidence N (%)* | *Primary site N (%)* | *Histologic type N (%)* | *Other items[b] N (%)* |
| 140–149, 160–161 | Mouth, pharynx and larynx | 12 | 4 (33) | 2 (17) | 1 (8) | 1 (8) | 0 (0) |
| 150–159 | Gastro-intestinal tract | 33 | 6 (18) | 3 (9) | 3 (9) | 0 (0) | 0 (0) |
| 162–165 | Lung and mediastinum | 32 | 1 (3) | 0 (0) | 0 (0) | 0 (0) | 1 (3) |
| 173 | Skin | 11 | 0 (0) | 0 (0) | 0 (0) | 0 (0) | 0 (0) |
| 174–175 | Breast | 29 | 3 (10) | 1 (3) | 0 (0) | 1 (3) | 1 (3) |
| 179–184 | Female genital organs | 21 | 2 (10) | 0 (0) | 2 (10) | 0 (0) | 0 (0) |
| 185–187 | Male genital organs | 15 | 0 (0) | 0 (0) | 0 (0) | 0 (0) | 0 (0) |
| 188–189 | Urinary tract | 16 | 4 (25) | 3 (19) | 0 (0) | 0 (0) | 1 (6) |
| 169, 196 | Lymphatic/haematopoetic | 8 | 3 (37) | 0 (0) | 0 (0) | 1 (13) | 2 (25) |
| 199 | Primary tumour unknown | 9 | 5 (56) | 1 (11) | 5 (56) | 0 (0) | 0 (0) |
| other | Other | 4 | 1 (25) | 0 (0) | 0 (0) | 1 (25) | 0 (0) |
| 140–199 | Total | 190 | 29 (15) | 10 (5) | 11 (6) | 4 (2) | 5 (3) |

[a]Records with one or more major disagreement in the items. Because some records have more than one major disagreement the total of the row can be higher than the stated total in this column. [b]Date of birth, gender, laterality and behaviour code.

study focused on disagreements in primary site. The number of major disagreements (11 out of 200 cases) was quite similar to our study, despite the use of other criteria. Lapham and Waugh reported a considerable number of disagreement with respect to malignant lymphomas. They attributed this to the fact that the lymphoma chapter of the ICD-9 has become outdated. In our study the malignant lymphomas were not especially prone to disagreements with respect to histologic type. We ascribe this to the fact that the Netherlands Cancer Registry uses the ICD-0 and has developed special codes for the histology of malignant lymphomas (Otter, 1989).

The quality of cancer registry data has received increasing attention in recent years, but only relatively few studies on this issue have been published. Those studies are in general focused on 'completeness' or 'accuracy' (Hilsenbeck, 1990). The studies on accuracy are, in fact, reproducibility studies. In this study we compared data from the source (the clinicians) with the data as abstracted and coded by registration personnel. The data from the clinicians cannot be used as a golden standard, because clinicians and registration personnel collect data with a different perspective. Whereas clinicians use their data for making the decisions on treatment and prognosis, registration personnel are trained to accomplish uniformity with the help of strict coding rules. Some clinicians did supply information with less than required detail, because the more detailed information was not directly relevent for their daily clinical practice.

Another possible reason for differences in information are differences in knowledge of the medical background of the patient. Registration personnel have to rely on the information in the clinical files, which is sometimes not fully complete. The clinician has more direct information on the patient and his disease, but has less knowledge of the coding rules of the cancer registry. In general, the information supplied by clinicians does agree rather well with the information abstracted and coded by registration personnel.

It can therefore be concluded that registration personnel, as trained and supported in the Netherlands cancer registries, are capable of collecting data for the cancer registry with a high degree of accuracy and reliability. However, coding rules for unknown primary malignancies ought to be improved and in some aspects the registration personnel need more training.

The authors want to express their gratitude to the following clinicians, who participated in this study: Dr C.G.M.I. Baeten, C. van de Beek, Dr J.M.H. van der Beek, P.J.M.J. Bessems, Dr L.R.H.I. Cuypers, Dr K.P.J. Delaere, W.A. van Deurzen, Dr L.G.J.B. Engels, J.T.H. Hellebrand, P.S.G.J. Hupperets, Dr M.R. von Meyenfeldt, W.A. Prins, Dr J. van der Putten, J.A. van der Snoek, Dr J.E.G.M. Stoot, H.J.G. Stroeken, I. Utama, Dr G.P.M. ten Velde, J. de Vries and Dr J.A.M.J. Wils; and to Dr R. Otter for her valuable comments.

### References

BERG, J.W. (1982). Morphologic classification of human cancer. In *Cancer Epidemiology and Prevention.* Schottenfeld, D. & Fraumeni, J.F. pp. 74–89. W.B. Saunders Company, Philadelphia.

CENTRALIZED CANCER PATIENT DATA SYSTEM (1985). Definitions of major and minor disagreements and standards for reabstracting. In *Quality control for cancer registries.* Hilsenbeck, S.G., Glaefke, G.S., Feigl, P. *et al.* pp. 127–132. National Institutes of Health, Government Printing Office: Bethesda Md.

HILSENBECK, S.G. (1990). Quality Control Practices in Centralized Tumor Registries in North America. *J. Clin. Epidemiol.*, **43**, 1201–1212.

INTERNATIONAL CLASSIFICATION OF DISEASES FOR ONCOLOGY (1976). World Health Organisation: Geneva.

LAPHAM, R. & WAUGH, N.R. (1992). An audit of the quality of cancer registration data. *Br. J. Cancer*, **66**, 552–554.

OTTER, R. (1989). *Non-Hodgkin's Lymphoma in a population-based registry.* pp. 165–188. University of Leiden: Leiden (thesis).

PARKIN, D.M., MUIR, C.S., WHELAN, S.L., GAO, Y.T., FERLAY, J. & POWELL, J. (1992). *Cancer Incidence in Five Continents, Volume VI.* IARC Scientific Publications No. 120. International Agency for Research on Cancer, Lyon.

ROBLES, R.C., MARRETT, L.D., CLARKE, E.A. & RISCH, H.A. (1988). An application of capture-recapture methods to the estimation of completeness of cancer registration. *J. Clin. Epidemiol.*, **41**, 495–501.

SCHOUTEN, L.J., VAN DEN BRANDT, P.A. & JAGER, J.J. (1992*a*). Cancer Incidence in the province of Limburg, the Netherlands. *Eur. J. Cancer*, **28A**, 1752–1755.

SCHOUTEN, L.J., VAN DEN BRANDT, P.A., JAGER, J.J., SMEETS, L.B. (1992*b*). Cancer Incidence in Maastricht, the Netherlands, 1986–1988. In *Cancer Incidence in Five Continents*, Volume VI. Parkin, D.M., Muir, C.S., Whelan, S.L., Gao, Y.T., Ferlay, J. & Powell, J. (eds.). IARC Scientific Publications No. 120. International Agency for Research on Cancer, Lyon.

SKEET, R.G. (1991). The Thames Cancer Registry. In *Cancer Registration: Principles and Methods.* Jensen, O.M., Parkin, D.M., MacLennan, R., Muir, C.S. & Skeet, R.G. (eds.) pp. 237–245. IARC Scientific Publications No. 95. International Agency for Research on Cancer: Lyon.

STORM, H.H. (1991). The Danish Cancer Registry, a self-reporting national cancer registration system with elements of active data collection. In *Cancer Registration: Principles and Methods.* Jensen, O.M., Parkin, D.M., MacLennan, R., Muir, C.S. & Skeet, R.G. (eds.) pp. 220–236. IARC Scientific Publications No. 95. International Agency for Research on Cancer: Lyon.